# Supplementary material for: Development of Genetic Tools in Glucoamylase-Hyperproducing Industrial Aspergillus niger Strains
Source: Biology (Basel). 2022 Sep 24;11(10):1396. doi: 10.3390/biology11101396 (PMC9599018; doi:10.3390/biology11101396)
Supplement: Supplementary file 1 [file biology-11-01396-s001.zip › biology-1894923-supplementary.pdf]

Supplementary Materials:

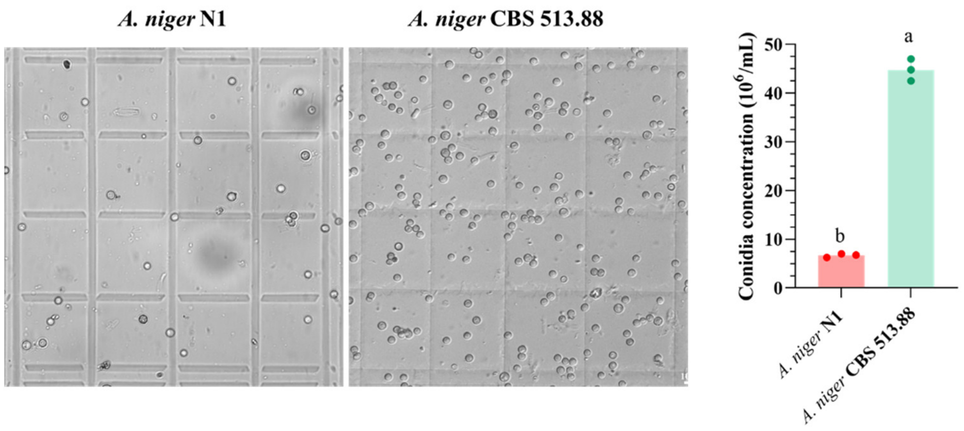

Figure S1. The number of conidia for *Aspergillus Niger* N1 and CBS 513.88. The number of conidia in each 4 × 4 square corner areas the hemocytometer of *A. niger* N1 (left image), The number of conidia in each 4 × 4 square corner areas of the hemocytometer of *A. niger* CBS 513.88 (middle image), column diagram of the number of conidia of *Aspergillus Niger* N1 and CBS 513.88 (right image). The left picture Data represent mean ± SD, Bars with different letters are statistically significant (Tukey's HSD, P < 0.05).

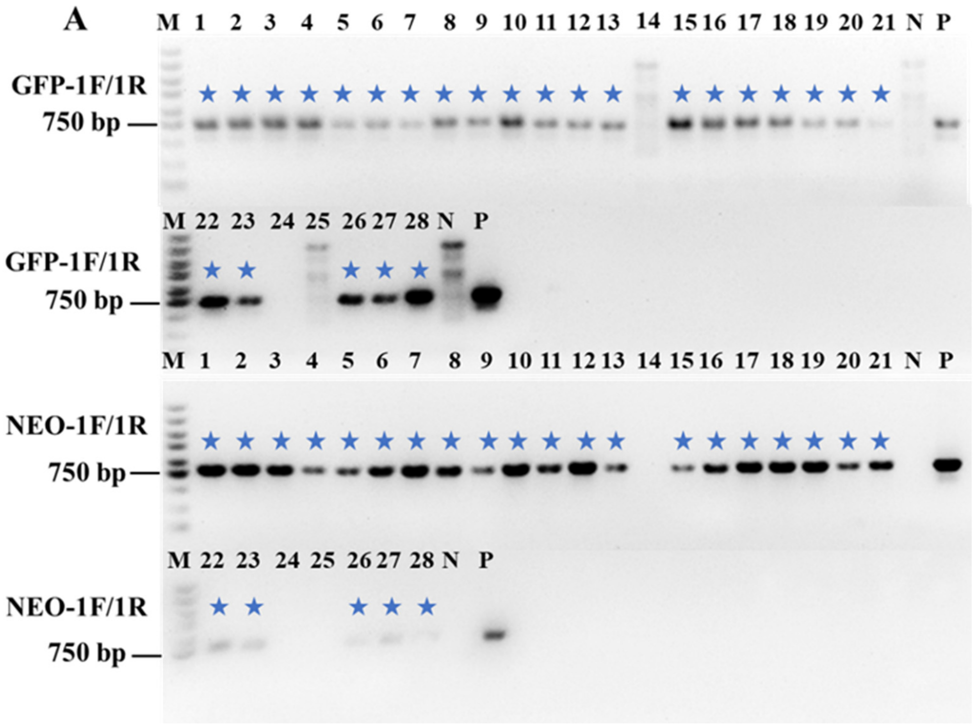

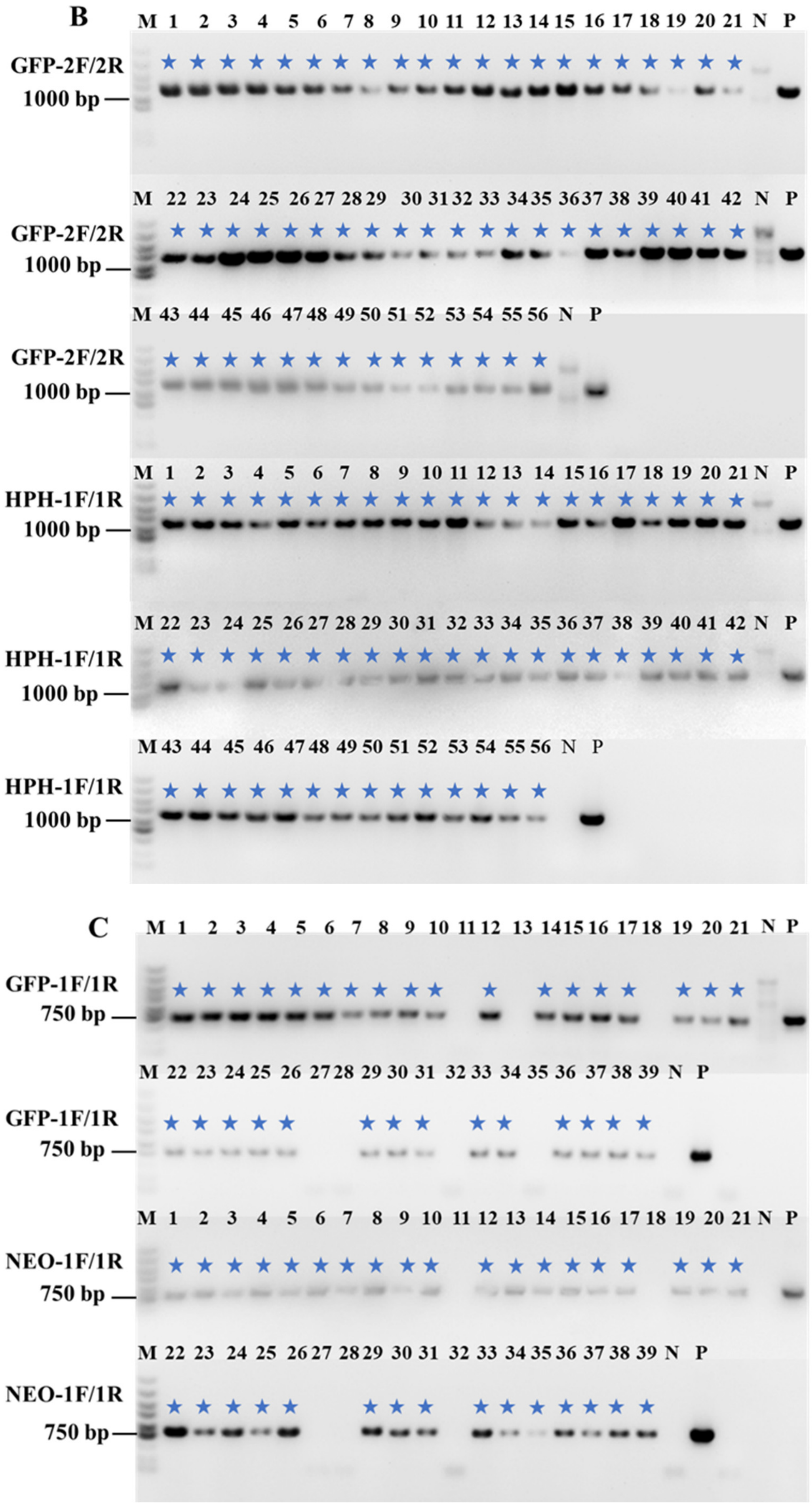
B
M 1 2 3 4 5 6 7 8 9 10 11 12 13 14 15 16 17 18 19 20 21 N P
GFP-2F/2R
1000 bp
M 22 23 24 25 26 27 28 29 30 31 32 33 34 35 36 37 38 39 40 41 42 N P
GFP-2F/2R
1000 bp
M 43 44 45 46 47 48 49 50 51 52 53 54 55 56 N P
GFP-2F/2R
1000 bp
M 1 2 3 4 5 6 7 8 9 10 11 12 13 14 15 16 17 18 19 20 21 N P
HPH-1F/1R
1000 bp
M 22 23 24 25 26 27 28 29 30 31 32 33 34 35 36 37 38 39 40 41 42 N P
HPH-1F/1R
1000 bp
M 43 44 45 46 47 48 49 50 51 52 53 54 55 56 N P
HPH-1F/1R
1000 bp
C
M 1 2 3 4 5 6 7 8 9 10 11 12 13 14 15 16 17 18 19 20 21 N P
GFP-1F/1R
750 bp
M 22 23 24 25 26 27 28 29 30 31 32 33 34 35 36 37 38 39 N P
GFP-1F/1R
750 bp
M 1 2 3 4 5 6 7 8 9 10 11 12 13 14 15 16 17 18 19 20 21 N P
NEO-1F/1R
750 bp
M 22 23 24 25 26 27 28 29 30 31 32 33 34 35 36 37 38 39 N P
NEO-1F/1R
750 bp

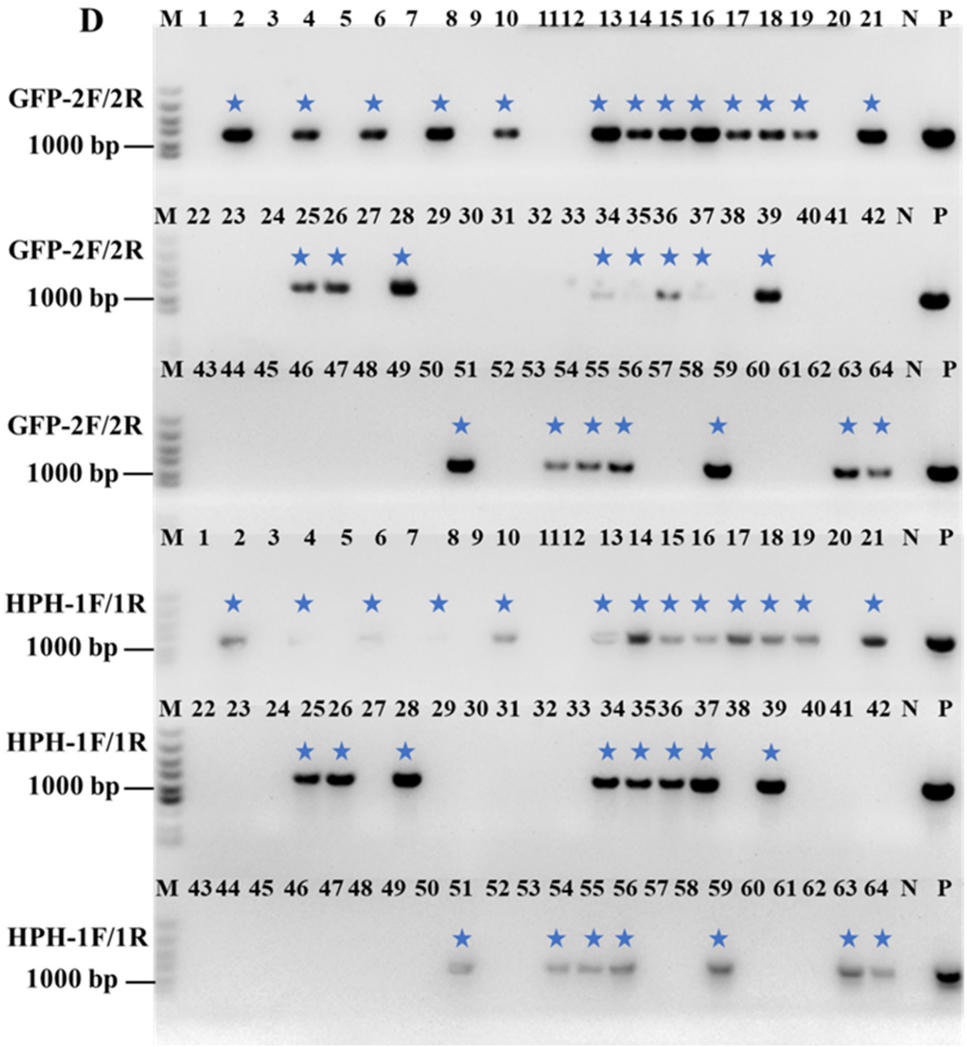

Figure S2. PCR validation of transformants of *A. niger* strains N1 and O1. (A) PCR validation with primers GFP-1F/1R (expected product of 720 bp) and NEO-1F/1R (expected product of 795 bp) for transformants of *A. niger* N1 generated using PMT. (B) PCR validation with primers GFP-2F/2R (expected product of 1253 bp) and HPH-1F/1R (expected product of 1201 bp) of transformants of *A. niger* N1 generated using AMT. (C) PCR validation with primers GFP-1F/1R (expected product of 720 bp) and NEO-1F/1R (expected product of 795 bp) of transformants of *A. niger* O1 generated using PMT. (D) PCR validation with primers GFP-2F/2R (expected product of 1253 bp) and HPH-1F/1R (expected product of 1201 bp) of transformants of *A. niger* O1 generated using AMT. M: DM5000 DNA ladder. N: Negative control, P: positive control. Asterisks indicate positive transformants.

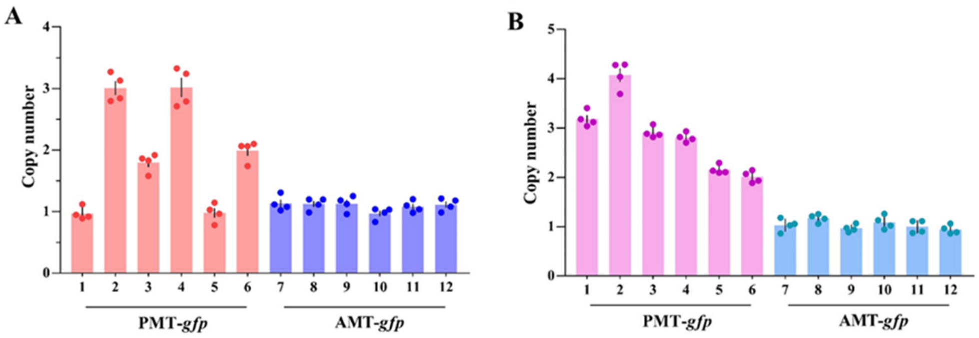

Figure S3. Assay of *gfp* gene copy number in mutants by RT-qPCR. (A) PMT-*gfp*: Transformants of *A. niger* N1 generated using PMT; AMT-*gfp*: transformants of *A. niger* N1 generated using AMT. (B) PMT-*gfp*: Transformants of *A. niger* O1 generated using PMT; AMT-*gfp*: transformants of *A. niger* O1 generated using AMT. Data represent mean ± SD from four replicates, Bars marked with different letters are statistically significantly different (Tukey's HSD, $P < 0.05$).

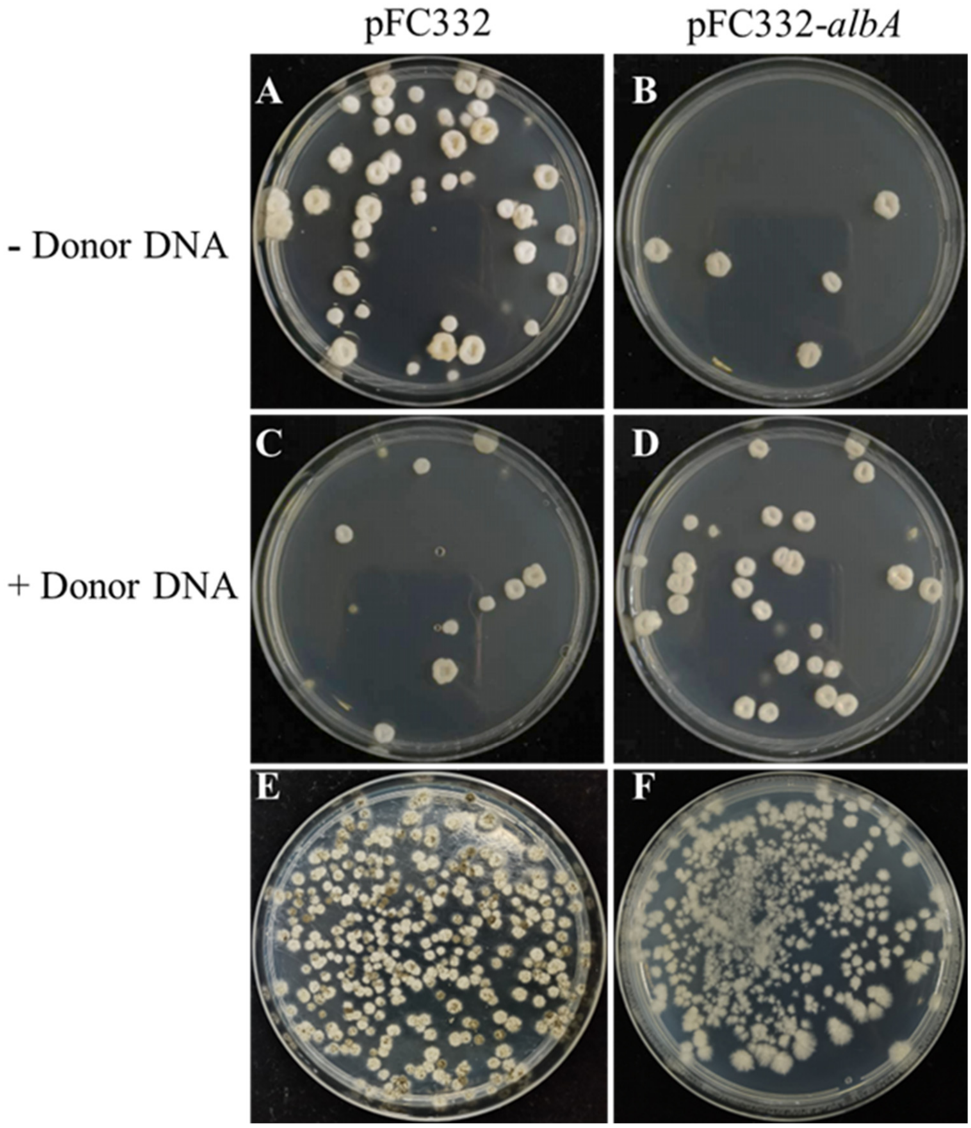

Figure S4. PMT of *A. niger* strain N1 targeting the *albA* gene by CRISPR–Cas9 system. Protoplasts were transformed with a pFC332-based vector, either with an sgRNA expression cassette (pFC332-*AnalbA*; B and D) or without any sgRNA expression cassette (pFC332; A and C), and were grown on bottom medium containing 50 μg/mL hygromycin B. A knockout-repair DNA fragment was either omitted (A and B) or added (C and D) to the protoplasts, in addition to the plasmid. (E) Streak of a single *albA*-knockout transformant taken from the plate shown in C, and (F) of a transformant taken from the plate shown in D.

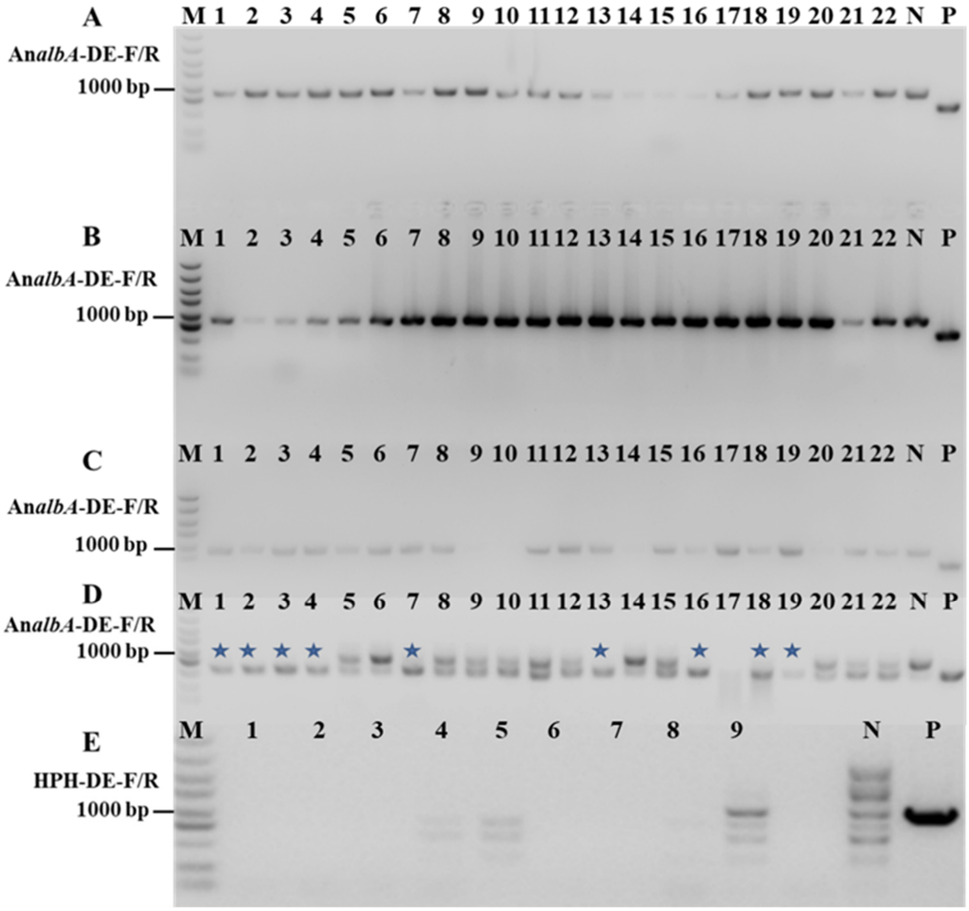

Figure S5. PCR validation of *albA*-knockout transformants. (A–D) Twenty-two transformants were randomly selected from the plates shown in Figure S3A, B, C and D for PCR identification using primers *AnalbA*-DE-F/R (expected product of 560 bp). Figure S3A correspond to Fig S4A, S3B to S4B, S3C to S4C, and S3D to S4D. N: Genomic DNA from A. *niger* strain N1 as a negative control (expected product of 856 bp); P: positive control (expected product of 560 bp). (E) PCR validation of the loss of the hygromycin-resistance gene from nine *albA*-knockout strains using primers HPH-DE-F/R. N: Genomic DNA from *A. niger* strain N1 as a negative control; P: positive control (expected product of 1006 bp). Asterisks indicate positive transformants.

Table S1. Sequences of primers used in PCR.

Name	Primer sequence (5′-3′)	Notes
Primer-1F	TGGCTGACTTGAAGTAATCTC	Cloning GFP expression cassette
Primer-1R	GCAAGGCGATTAAGTTGGGTA	Cloning GFP expression cassette
GFP-1F	ATGGTGAGCAAGGGCGAGGAGCTGT	PCR detection of GFP
GFP-1R	TTACTTGTACAGCTCGTCCATGCCGAGA	PCR detection of GFP
NEO-1F	ATGATTGAACAAGATGGATTGCACGCAG	PCR detection of NEO
NEO-1R	TCAGAAGAACTCGTCAAGAAGGCGATAGAAG	PCR detection of NEO
GFP-2F	TTACTTGTACAGCTCGTCCATGCCG	PCR detection of GFP
GFP-2R	CCAAGCATGGGCAGTGAGCGGCTATAC	PCR detection of GFP
HPH-1F	TCGACGTTAACTGATATTGAAGGAG	PCR detection of HPH
HPH-1R	GCTCTGATAGAGTTGGTCAAGACC	PCR detection of HPH
gfp-RT-F	CACCATCTTCTTCAAGGAC	RT-qPCR of *gfp*
gfp-RT-R	GGCTGTTGTAGTTGTACTC	RT-qPCR of *gfp*
actin-RT-F	ATTGGTATGGGTCAGAAG	RT-qPCR of Anactin
actin-RT-R	GAGTTCATTGTAGAAGGTG	RT-qPCR of Anactin
Probe-F	CTTGATGCCGTTCTTCTGCTTG	Probe of Southern blotting
Probe-R	CCTCGTGACCACCCTGACCTAC	Probe of Southern blotting
AnalbA-5F	ACACAGGAAACAGCTATGACCATGATTACG	5' flanking fragment
AnalbA-5F	AATTCAAGCGGAAACTGAAGGATACTACTGG	5' flanking fragment
AnalbA-5R	TTATCATCATACAGATGTGGCGCATGG	5' flanking fragment
AnalbA-5R	AGTCCACCAGTTCTCGAACTCGAA	5' flanking fragment
AnalbA-3F	TTCGAGTTCGAGAACTGGTGGACTCC	3' flanking fragment
AnalbA-3F	ATGCGCCACATCTGTATGATGATAA	3' flanking fragment
AnalbA-3R	GTGCCAAGCTTGCATGCCTGCAGGTCGACTCTAGA	3' flanking fragment
AnalbA-3R	TCAACGCTCACACTGGGGCCGCTAAATTTG	3' flanking fragment
AnalbA-DE-F	CATTGACATGCATCTACCAATTGGG	PCR detection of *AnalbA*
AnalbA-DE-R	CTACTCGAGATCGGAAACAATTTGC	PCR detection of *AnalbA*
HPH-DE-F	ATGAAAAAGCCTGAACTCACCGCGA	Loss of HPH
HPH-DE-R	GAGTGCTGGGGCGTCGGTTTCCACT	Loss of HPH
